# A Valuable Table

**Published:** 1864-04

**Authors:** 


**A Valuable Table.**—I notice in the *Farmer* of July 26 an article under the above caption, which would be valuable if it was correct; but I find so much discrepancy in it that I am constrained to write.

When I was a boy I learned from Adams' old arithmetic that 268.8 cubic inches make a gallon dry measure, and on that supposition, the first box, 24 by 16 by 28 inches, said to contain five bushels or one barrel, is correct, if you call 40 gallons a barrel; but that is not the way we reckon barrels here. No matter—it is the boxes we are after now: all correct, so far.

But the second box, said to contain half as much as the first, is of the same length and breadth, and should be 14 inches deep instead of 12 inches.

The third box, 26 by 15.8 by 8 inches, said to contain one bushel, does contain over a bushel and a half.

The fourth box, 12 by 11.2 by 8 inches, said to contain one peck, does contain just half a bushel.

The fifth box, 8 by 8 by 4.2 inches, said to contain a gallon, is correct.

The sixth box, 4 by 8 by 4.8 inches, said to contain a half gallon, is 19.2 cubic inches too large.

The seventh box, 4 by 4 by 4.1, said to contain a quart, is 1.6 cubic inches too small.

Now I have my hand in, if you have room to spare, I should like to give a simple rule to ascertain the correctness of grain measures in the form commonly used for half-bushels, pecks, etc.—that is, the round or circular form.

First, to find the area of any circle, multiply the square of its diameter by .7854, that is the decimal form of 7854-10,000, and the product will be the answer. And now for the half-bushel.

Measure the diameter carefully in inches and fractions of an inch, (a carpenter's square will answer all practical purposes, but the Gunter's scale is better, because it gives the fractions in decimal form,) then multiply its square by 7854, as directed above, and you have the number of square inches checked right out on the half-bushel bottom, by which divide the number of cubic inches in half a bushel, and the quotient will be the required depth in inches and fractions of an inch. Now measure perpendicularly, and if not correct, cut down the top or move the bottom outward or inward.

